# The reliability of methods to estimate the number and size of human motor units and their use with large limb muscles

**DOI:** 10.1007/s00421-018-3811-5

**Published:** 2018-01-22

**Authors:** M. Piasecki, A. Ireland, J. Piasecki, D. W. Stashuk, J. S. McPhee, D. A. Jones

**Affiliations:** 1 School of Healthcare Science, Manchester Metropolitan University, Manchester, M15GD UK; 2 Department of Systems Design Engineering, University of Waterloo, Ontario, N2L 3G1 Canada

**Keywords:** Motor unit number estimation, Electromyography, Skeletal muscle, Vastus lateralis

## Abstract

**Purpose:**

Current methods for estimating muscle motor unit (MU) number provide values which are remarkably similar for muscles of widely differing size, probably because surface electrodes sample from similar and relatively small volumes in each muscle. We have evaluated an alternative means of estimating MU number that takes into account differences in muscle size.

**Methods:**

Intramuscular motor unit potentials (MUPs) were recorded and muscle cross-sectional area (CSA) was measured using MRI to provide a motor unit number estimate (iMUNE). This was compared to the traditional MUNE method, using compound muscle action potentials (CMAP) and surface motor unit potentials (sMUPs) recorded using surface electrodes. Data were collected from proximal and distal regions of the vastus lateralis (VL) in young and old men while test–retest reliability was evaluated with VL, tibialis anterior and biceps brachii.

**Results:**

MUPs, sMUPs and CMAPs were highly reliable (*r* = 0.84–0.91). The traditional MUNE, based on surface recordings, did not differ between proximal and distal sites of the VL despite the proximal CSA being twice the distal CSA. iMUNE, however, gave values that differed between young and old and were proportional to the muscle size.

**Conclusion:**

When evaluating the contribution that MU loss makes to muscle atrophy, such as in disease or ageing, it is important to have a method such as iMUNE, which takes into account any differences in total muscle size.

## Introduction

Loss of muscle mass and strength are primary features of ageing and while there may be a number of causes, including atrophy or the reduction in specific force of individual fibres, there is good evidence that a reduction in the number of fibres (Lexell et al. 1988) as a consequence of the loss of motor neurons (Tomlinson and Irving 1977) plays an important part in the ageing process. Research techniques are needed to elucidate the underlying mechanisms and possible measures to alleviate these changes. Although motor unit numbers have been determined in small peripheral muscles (Boe et al. 2006; Hourigan et al. 2015), it is important to monitor changes in large proximal muscle groups that are key to changes in mobility.

Current methods for estimating numbers of motor units in larger muscles, often referred to as “motor unit number estimates” (MUNE), rely on comparing the average size of surface recorded motor unit potentials (sMUPs) with a maximal electrically stimulated compound muscle action potential (CMAP). The rationale for this is that the size of the maximal CMAP is a measure of the electrical activity of the whole muscle and dividing this by the size of an average sMUP provides an estimate of the number of MUs (Brown et al. 1988).

There are a range of MUNE methods which differ largely in the way the average sMUP is obtained, the most commonly used in recent years is ‘spike-triggered averaging’ (STA) in which individual sMUPs are identified and averaged using a trigger from an intramuscular needle electrode. This was first proposed by Brown et al. (1988) and further developed with improved recording and analytical methods (Daube 2006; Bromberg 2007; Gooch et al. 2014) that have been used to characterize MU loss in disease (Gooch et al. 2009; Allen et al. 2015) and ageing (McNeil et al. 2005; Power et al. 2012; Piasecki et al. 2015).

The STA methodology has been used to produce MUNE values for a variety of muscles and it is surprising to see that the estimated number of reported motor units varies very little despite considerable differences in muscle size. For instance, MUNE values from 138 for the first dorsal interosseous (Boe et al. 2006) to around 200 for the vastus lateralis (Piasecki et al. 2016b) have been reported. In addition, some concern exists about the application of MUNE techniques to small muscles because cross talk from nearby muscles may increase the CMAP value (Kawamura et al. 2013). With large muscles, the CMAP may not properly represent the whole muscle and the sMUPs may only reflect superficial motor units. There is a need to develop new techniques to study motor unit characteristics of large proximal muscle groups that are prone to age-related wasting and are key for mobility.

Given these doubts about MUNE based on surface EMG data, we have previously proposed an alternative method referred to as intramuscular MUNE (iMUNE) (Piasecki et al. 2016a, b). This approach is based on the fact that the size of a MUP recorded from an intramuscular electrode is proportional to the number of fibres contributing to it (Nandedkar et al. 1988) and will, therefore, also be proportional to the cross-sectional area (CSA) of muscle occupied by that unit. Therefore, the ratio of the mean MUP size to the CSA of the muscle can provide an index representing the total number of motor units in that muscle.

The purpose of the present study was to compare the iMUNE with the more commonly used MUNE method based on surface EMG with particular emphasis on the results obtained from proximal and distal portions of the vastus lateralis, which are of very different size, as well as examining the reliability of the methods when used in different muscle groups.

## Methods

### Participants and recruitment

Participants were recruited from amongst University staff and students as well as the local community. Volunteers were included if they were male, habitually physically active but not competing in sports at a regional level or above, and with no history of metabolic, orthopaedic or neuromuscular disease. The study was approved by the University Research Ethics Committee and was conducted in accordance with the Declaration of Helsinki. All participants provided informed consent in writing.

### Anthropometry measures

**Fig. 1:**
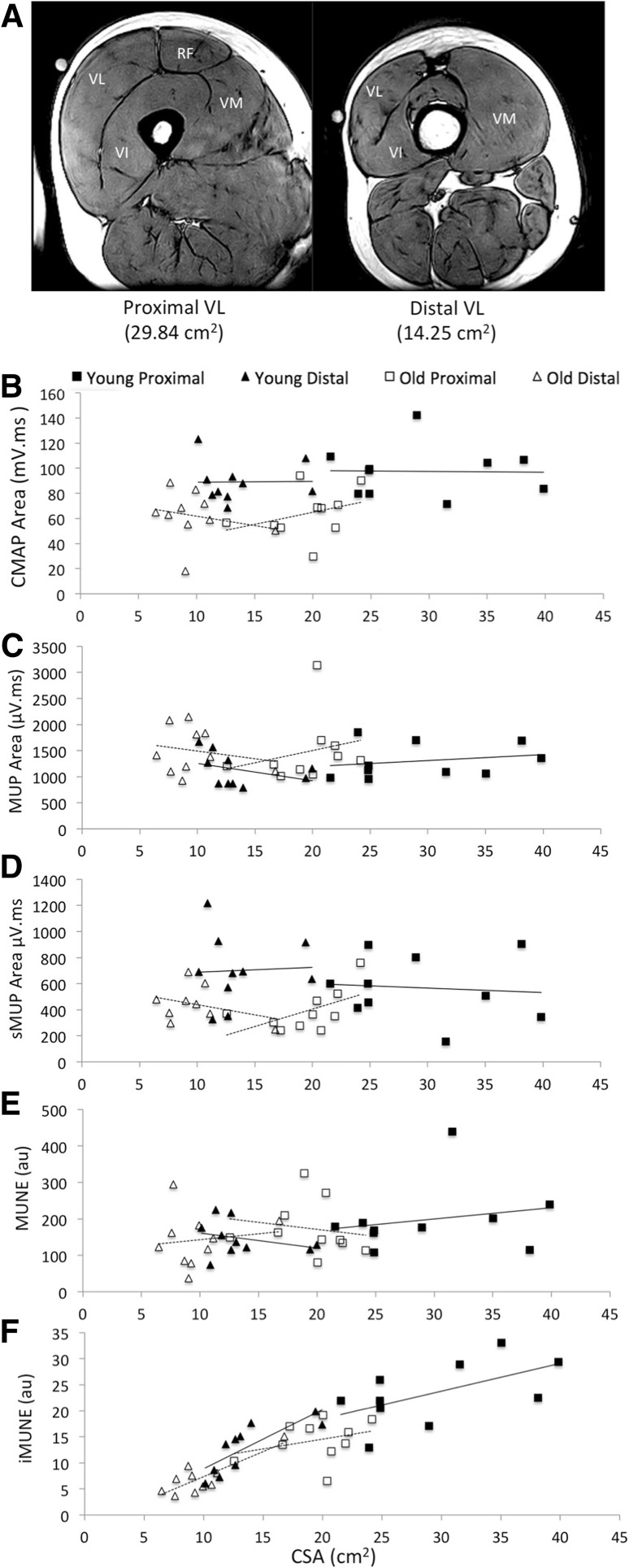
**a** Example MRI images of proximal and distal location of VL from a young participant. Circular markers indicate site of needle insertion. Values indicate CSA at each site from ten participants. **b**–**f** Neuromuscular characteristics and CSA recorded from proximal and distal motor points of the VL in young and old participants. Solid trendlines relate to young participants, dashed trendlines relate to old participants. *MUP* motor unit potential, *sMUP* surface motor unit potential, *CMAP* compound muscle action potential, *MUNE* motor unit number estimate, *iMUNE* intramuscular motor unit estimate, *VL* vastus lateralis, *RF* rectus femoris, *VM* vastus medialis, *VI* vastus intermedius

Magnetic resonance imaging (MRI) was used to measure muscle CSA of the VL, TA and BB using a T1-weighted turbo 3D sequence on a 0.25-T G-Scan (Esaote, Genoe, Italy). The scanning coil was positioned over the motor point, and contiguous transverse-plane slices of 6 mm thickness were collected along a 14-cm length with the participant lying rested and supine. For the comparison of proximal and distal sites of the VL, the process was repeated at each motor point. Fat and skin thickness was also calculated using the same images. All images were analysed off-line using Osirix imaging software (OsiriX medical imaging, OsiriX, Atlanta, USA) and for all three muscles the CSA was recorded at the motor point (example images shown in Fig. 1a) (Maden-Wilkinson et al. 2014).

### Identification of motor points

The motor point was defined as the site of muscle that produced the largest visible twitch from the smallest stimulating current (Botter et al. 2011), and each was identified using low-intensity percutaneous electrical stimulations. A cathode probe (Medserve, Daventry, UK) was placed over the skin and a constant current simulator (DS7AH Digitimer, Welwyn Garden City, Hertfordshire, UK) was set to a compliance voltage of 400 vs with a 50-µs pulse width and the current varied, but was typically around 8 mA for all muscles. For VL, the distal motor point was located along the centerline of the VL around 90 mm from the lateral femoral condyle. The proximal motor point was located on a similar line around 220 mm from the lateral femoral condyle (Becker et al. 2010). For BB, the motor point was located approximately 155 mm distal of the coracoid process and 25 mm medial of the long and short head division (Moon et al. 2012). The TA motor point was located approximately 120 mm from the tibial head, over the muscle belly (Bowden and McNulty 2012).

### EMG recordings

The skin over each motor point was prepared by shaving and cleansing with an alcohol swab. The active sEMG electrode (disposable self-adhering Ag–AgCl electrodes; 95 mm^2^_,_ Ambu Neuroline, Baltorpbakken, Ballerup, Denmark) was placed over the motor point. The reference sEMG electrode was fixed over the patella tendon for VL and TA, and over the forearm extensor tendon for BB. Intramuscular EMG signals were recorded using disposable concentric needle electrodes with a recording area at the bevel of 0.07 mm^2^ (Model N53153; Teca, Hawthorne, NY). A shared ground electrode for surface and intramuscular EMG signals was placed over the patella for VL and TA, and over the olecranon for BB. Two CED 1902 amplifiers (Cambridge Electronics Design Ltd, Cambridge, UK) were used to bandpass sEMG and iEMG signals at 5 Hz to 5 KHz and 10 Hz to 10 KHz, respectively. Signals were digitized with a CED Micro 1401 data acquisition unit (Cambridge Electronic Design). The sEMG signals were sampled at 10 KHz and the iEMG signals at 25 KHz. Both EMG signals and the force signal were recorded and displayed in real-time via Spike2 software (v8.01). Data were stored for off-line analysis.

### Experimental procedures

Percutaneous stimulation of the femoral, common peroneal or musculocutaneous nerve was used to acquire the maximal CMAP of VL, TA and BB muscles, respectively, using a manually triggered stimulator (model DS7AH; Digitimer, Welwyn Garden City, Hertfordshire, UK). The current was increased incrementally with each stimulation, until the CMAP no longer increased in size, which generally occurred between 100 and 200 mA. The current was then increased by 30 mA to ensure supra-maximal stimulation.

The participant was asked to relax the muscle and an intramuscular needle electrode was inserted at an angle to ensure the tip was beneath the active sEMG electrode and to a depth of 1.5–2 cm although somewhat less for smaller muscles. The needle position was adjusted to ensure the detection of MUPs with sharp rise-times. The participant was then instructed to perform a series of voluntary contractions at 25% MVC, each lasting 15 s with real-time visual feedback and approximately 30 s rest between contractions. After each series of contractions, the needle was relocated by withdrawing it by ~ 5 mm and rotating 180°, giving a total of between 4 and 6 contractions. Measurements were made at the two motor points of the VL and one motor point of the BB and TA.

### EMG signal decomposition and motor unit number estimation

Decomposition and analysis of EMG signals were performed with DQEMG software (Stashuk 1999; Piasecki et al. 2016a, b). Traditional MUNE values were obtained by dividing the size (area) of the mean sMUP into the size (area) of the electrically evoked maximal CMAP. The mean sMUP was estimated using the set of ensemble-averaged sMUPs estimated using the recorded sEMG signals and the firing times of the motor units sampled across the series of completed voluntary contractions. The iMUNE value was obtained by dividing the CSA (cm^2^) of a muscle into the mean MUP area (cm^2^/mV/ms), and is expressed as arbitrary units.

### Statistical analysis

The results are set out in accordance with the main objectives. To determine the degree to which physiological characteristics and MU parameters differ between different portions of the VL in young and old participants, measurements from proximal and distal sites of the VL were compared using a two-way ANOVA, with location and group as fixed factors. We have included minimal discussion of age differences because this was not the purpose of the present work and age differences have been reported previously (Piasecki et al. 2016a, b). To address the second objective, to determine the repeatability of MU parameters and MUNE values, correlation analysis was performed using the Pearson’s Product Moment correlation coefficient (*r*). The test–retest reliability was calculated as the mean of the differences between each pair of observations divided by the average of the two observations, expressed as a percentage. Data are presented as mean (SD). Statistical significance was accepted at *p* ≤ 0.05. Statistical analysis was performed using SPSS Version 23 (SPSS, Chicago, IL).

## Results

### Comparison of proximal and distal regions of VL

In a group of ten healthy young men (mean age 27 ± 5 years) and ten healthy older men (mean age 70 ± 3 years), iEMG and sEMG signals were recorded from the proximal and distal motor points of the VL at 25% MVC. General participant characteristics and MRI measurements of VL are shown in Table 1. There was no significant interaction between the effects of location and age in skin and subcutaneous tissue thickness, *F*(1, 36) = 0.175, *p* = 0.679, or VL CSA, *F*(1, 36) = 4.071, *p* = 0.052. Skin and subcutaneous tissue thickness did not differ between young and old, or at proximal and distal locations. Vastus lateralis CSA was larger at the proximal site, and larger in the young compared to the old (Table 1. Fig. 1).

**Table 1:** Participant characteristics for detailed assessments of vastus lateralis

	Young		Old			
Age (years)	27.3 (5.2)		70.3 (3.1)			
Height (cm)	176 (5.3)		173 (8.6)			
Weight (Kg)	73.2 (10.8)		74.9 (10.1)			

	Young		Old		Location	Age
MRI	Proximal	Distal	Proximal	Distal		
Fat + skin thickness (cm)	0.39 (0.07)	0.38 (0.06)	0.36 (0.17)	0.31 (0.18)	*0.632*	*0.616*
VL CSA (cm^2^)	29.8 (2.6)	14.3 (1.2)	19.5 (3.3)	9.7 (2.9)	< *0.0005*	< *0.0005*

Data are mean (SD). *p* values showing main effects of location and age are shown in italics in the final columns

*CSA* cross-sectional area, *VL* vastus lateralis

There was no significant interaction between the effects of location and age in MUP area *F*(1, 36) = 0.458, *p* = 0.503, CMAP area *F*(1, 36) = 0.318, p = 0.576, sMUP area *F*(1, 36) = 0.360, *p* = 0.552, MUNE *F*(1, 36) = 0.184, *p* = 0.670, or iMUNE *F*(1, 36) = 1.214, *p* = 0.278. Although CMAP differed between young and old, there was no difference between proximal and distal locations (Table 2; Fig. 1b) despite the twofold differences in CSA. Surface recorded sMUPs did not differ between the two sites (Fig. 1c) and consequently MUNE, calculated from surface recordings, did not differ between the two sites (Table 2). Areas of intramuscularly recorded MUPs were larger in the older men (*p* = 0.058) but did not differ between sites for either young or old (Table 2; Fig. 1c). Values of iMUNE, calculated from the intramuscularly recorded MUP and muscle CSA measured at the motor point, were significantly greater at the larger proximal compared to the distal site (Table 2).

**Table 2:** Neuromuscular electrophysiological characteristics recorded from proximal and distal motor points of vastus lateralis

	Young		Old		Location	Age
	Proximal	Distal	Proximal	Distal		
Number of MUs sampled	215	170	268	189		
MUP area (µV ms)	1302 (329)	1135 (403)	1477 (623)	1500 (437)	*0.609*	*0.058*
CMAP area (mV ms)	97.6 (20.5)	89.1 (16.1)	63.9 (19.1)	62.2 (19.4)	*0.403*	*< 0.0005*
sMUP area (µV ms)	499 (239)	653 (265)	390 (158)	441 (140)	*0.186*	*0.003*
MUNE	197 (92)	146 (47)	174 (74)	142 (42)	*0.085*	*0.540*
iMUNE	23.4 (6.1)	12.9 (4.9)	14.3 (3.9)	7.1 (3.3)	*< 0.0005*	*< 0.0005*

Recordings were made at the proximal and distal motor points of VL during contractions held at 25% MVC. Data are mean (SD). *P* values showing main effects of location and age are given in italics in the final columns

*MUP* motor unit potential, *sMUP* surface motor unit potential, *CMAP* compound muscle action potential, *MUNE* motor unit number estimate, *iMUNE* intramuscular motor unit estimate

### Repeatability

**Fig. 2:**
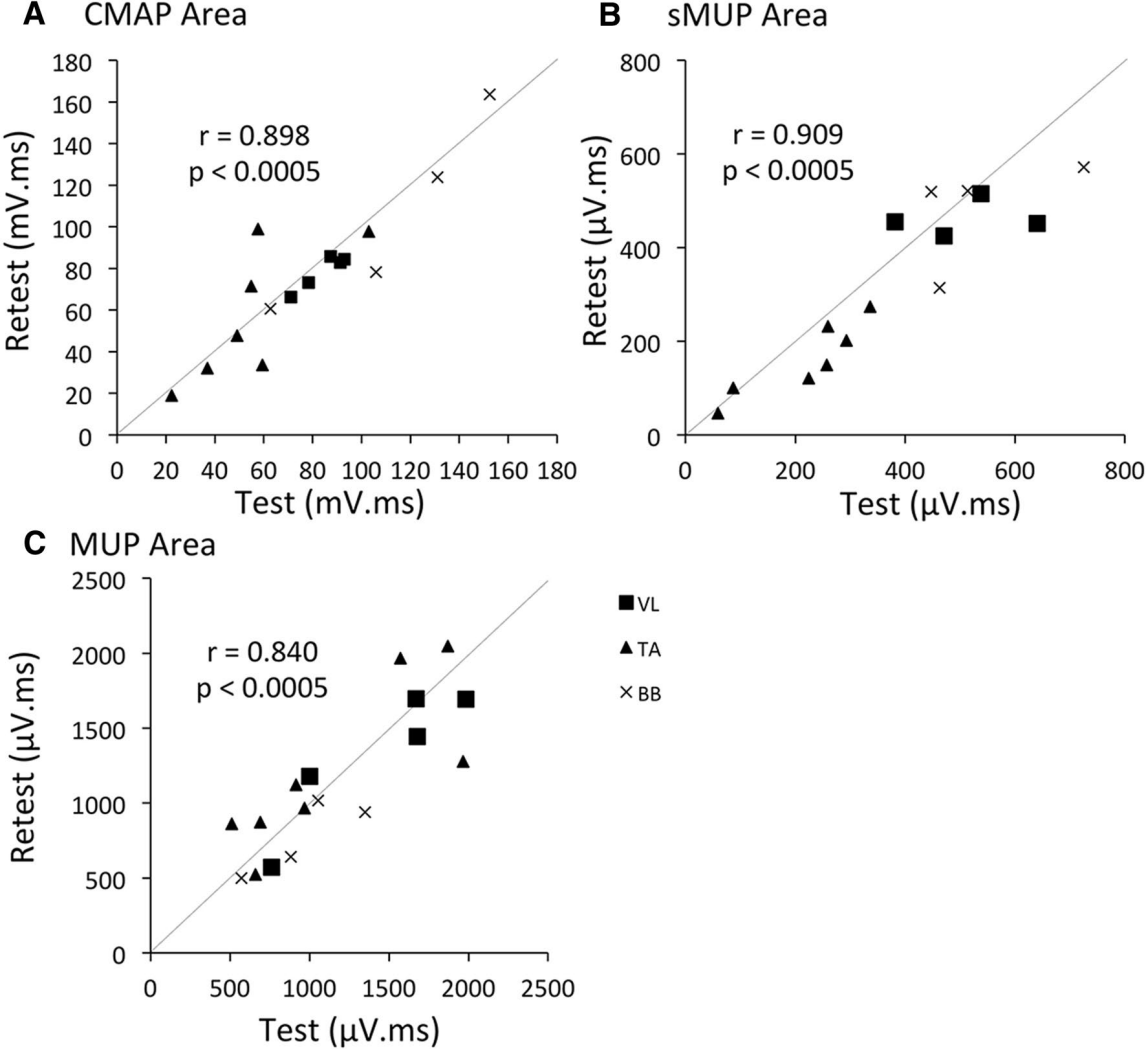
Repeatability of primary outcome measurements. Correlation coefficient of test and retest data; **a** Muscle mean MUP area, **b** CMAP area **c**, Muscle mean sMUP area. CMAP, compound muscle action potential; sMUP, surface motor unit potential; MUP, motor unit potential. Grey lines indicate line of identity (*y* = *x*)

Six young (mean age 31 ± 3) and three older men (mean age 72 ± 4) took part in this study, and test and retest data were collected from a total of 17 muscles, consisting of 5 VL, 8 TA and 4 BB. Participants were studied on two separate occasions separated by between 1 and 9 days. Data for the repeated measurements of the primary outcome variables, CMAP, muscle mean sMUP and muscle mean MUP areas are shown in Fig. 2.

**Fig. 3:**
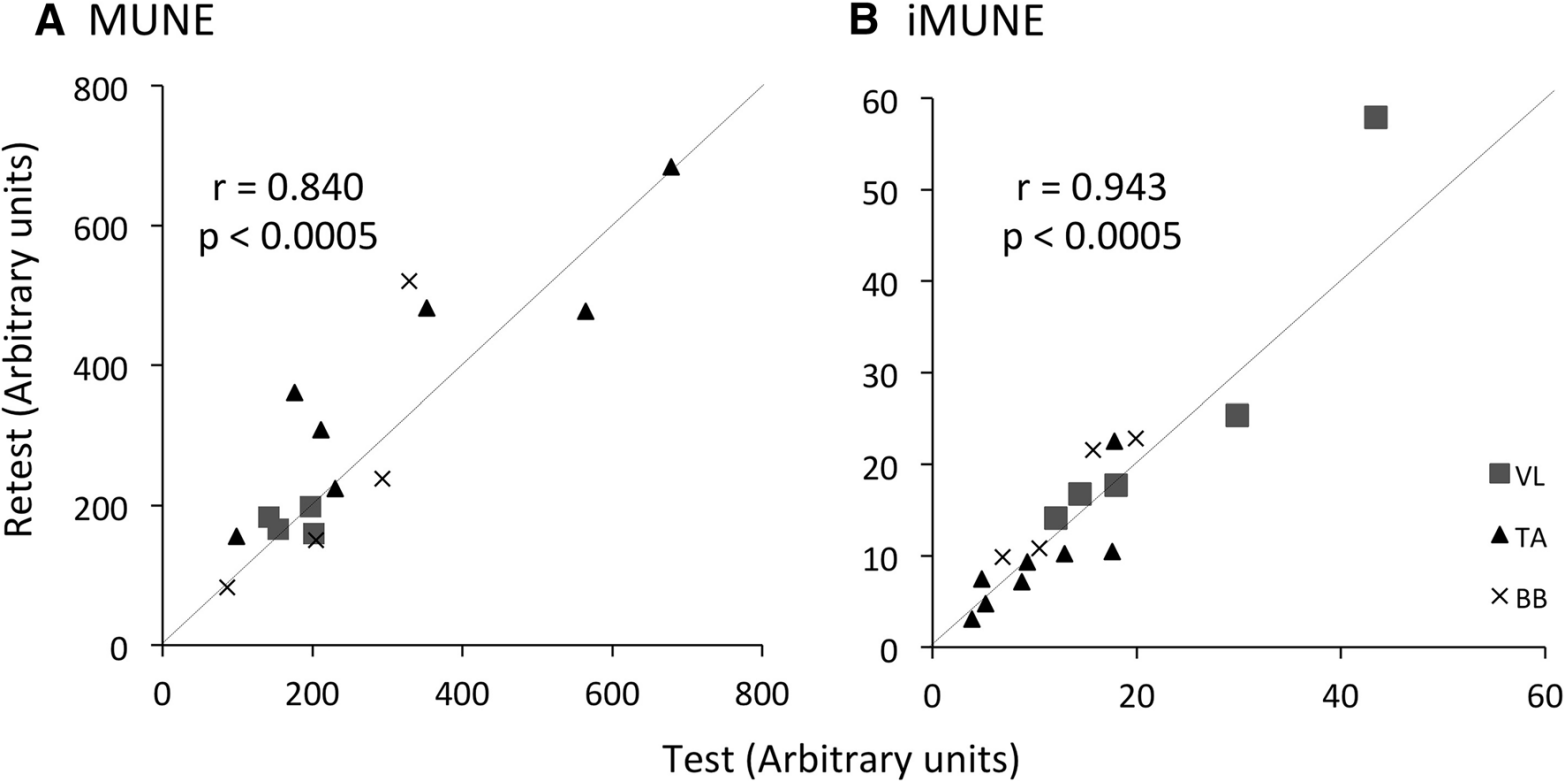
Repeatability of the derived MU number estimates. Correlation coefficient of test and retest data; **a** Motor unit number estimate, **b** Intramuscular motor unit number estimate. Grey lines indicate line of identity (*y* = *x*)

All measurements showed strong correlations between test and retest with *r* values from 0.84 to 0.909 (*p* < 0.0005). Mean percentage differences for CMAP, sMUP and MUP were 15.9, 15.1 and 4.4%, respectively, and were 15.7% for the MUNE and 7.5% for the iMUNE (Figs. 2, 3).

## Discussion

The loss of motor neurons is central to motor neuron disease and is likely to play a part in the ageing process, and it is very difficult to quantify the changes, especially for the larger proximal muscles where atrophy can limit mobility and quality of life. Methods for estimating numbers of motor units, and thus motor neurons, have been available for many years but recent advances in methodology and decomposition of complex needle and surface electrode signals have enabled a wider application of the methods. Even so, there have been few studies of the larger proximal muscles. Progress in understanding the ageing process in skeletal muscle would be greatly assisted by a method that provides an estimate of the number of motor units in the large muscles.

Estimates of motor unit number based on surface EMG recordings (MUNE) make the assumption that the CMAP represents the electrical activity of the whole muscle but the current study demonstrates that the CMAP is the same size for two portions of the VL which differ twofold in CSA (Tables 1, 2; Fig. 1). Consequently, the MUNE calculated for the proximal and distal regions was the same (Table 2), and this was true for both young and old muscle. The most obvious reason for this is that the surface electrodes sample from a fraction of the muscle which is similar in volume, a hemisphere with approximately a 2-cm radius (Barkhaus and Nandedkar 1994), and is not proportional to the size of the muscle at that point. Consequently for larger muscles, the deeper MUs are not fully represented by surface recordings (Muceli et al. 2015) because EMG signals are attenuated by overlying muscle, subcutaneous tissue and fat (Nordander et al. 2003). Thus, MUNE values tend to relate to the number of motor units within a given volume of muscle giving rise to relatively constant values rather than representing the total number of MUs within a muscle. This would also explain why MUNE values bear no relation to the size of a range of muscles (Table 3). There is also good reason to be cautious when applying MUNE to smaller muscles where the surface EMG signals, especially the CMAP, may be contaminated by electrical signals from adjacent muscles (Kawamura et al. 2013) leading to an overestimation of MUNE values.

**Table 3:** Motor unit number estimates reported using spike triggered averaging

Muscle	MUNE	
Vastus lateralis	195	Piasecki et al. (2016b)
Tibialis anterior	208	Piasecki et al. (2016a)
Tibialis anterior	187	Hourigan et al. (2015)
Tibialis anterior	150	McNeil et al. (2005)
Tibialis anterior	122^a^	Boe et al. (2009)
Biceps brachii	354	Power et al. (2012)
Biceps brachii	265^a^	Boe et al. (2006)
Upper trapezius	307^a^	Ives and Doherty (2012)
First dorsal interosseous	138^a^	Boe et al. (2006)

^a^The mean value from a test and retest

If, in large muscles, MUNE only gives an estimate of motor units in a given volume of muscle, one solution to the problem of dealing with muscles of different size would be to multiply the MUNE values by the CSA of the muscle, but there are potential problems with this approach. The first is that the volume of muscle sampled will probably differ from person to person depending on the amount of subcutaneous fat and conductivity of the skin, and the second is that motor units in the superficial layers of the muscle sampled by the surface electrodes may not be representative of the whole muscle.

The iMUNE method assumes that MUP size (area or amplitude give similar results) is a reflection of the number of fibres of a single MU within the recording limits of the electrode, and thus represents the CSA occupied. Dividing the CSA by MUP size, therefore, provides an index related to motor unit number in the whole muscle. Sampling from a number of different depths within the muscle also helps to minimize possible problems with a predominance of larger and smaller motor units at different depths within the muscle, as it has been reported that superficial MUs are larger than those located deeper in the VL (Knight and Kamen 2005).

It might be argued that in seeking differences between young and old it is sufficient to just measure CSA since across all of the subjects studied the difference in MUP size was not significant. However, for an individual subject, the iMUNE, which is the ratio of CSA to MUP size, can still be reflective of motor unit number. Furthermore, it is well documented that MUPs in old subjects are significantly larger compared with young when reporting data from larger cohorts of subjects (Hourigan et al. 2015; Piasecki et al. 2016a, b). Thus in general, the combination of smaller CSA and larger MUPs would have a greater effect on the iMUNE.

The second part of this study demonstrated that the EMG measures involving STA and DQEMG were highly repeatable with mean differences between test and retest of 4–15%, and regression coefficients from 0.84 to 0.91, respectively. This is the first study to include the reliability of measurements made on the large, proximal, VL. Similar correlations between test and retest have been reported for BB and FDI (Boe et al. 2006), thenar muscles (Boe et al. 2004), the TA (Boe et al. 2009), as well as the upper trapezius (Ives and Doherty 2012). Of the derived estimates of motor unit number, iMUNE was a slightly more repeatable measure than MUNE (Fig. 3b). The different muscle groups were assessed individually for repeatability, and although the small sample size precludes definitive statements, there was no suggestion that measurements of any one muscle group were more reliable than another.

Although current DQEMG techniques are a considerable advance on previous manual methods, it becomes difficult to reliably distinguish individual MUPs for contractions stronger than about 40% MVC, although this is muscle dependent. Consequently, both MUNE and iMUNE methods will sample motor units which are recruited at lower forces and it is well documented that both MUP and sMUP size increase with increasing force of contraction (Boe et al. 2005). Thus, the mean MUP or sMUP size will tend to be an underestimate of the true mean of the whole muscle and the calculated MUNE or iMUNE values will be over-estimates of the actual number within the whole muscle. It is clearly important, therefore, that measurements are made with a standardized contraction and most studies, including our own, have used 25% MVC. This does, however, require the MVC to be accurately determined and this may be difficult where subjects have problems with maximal activation of their muscles.

## Conclusions

EMG recordings with both surface and needle electrodes are reliable and repeatable. When surface recordings are used to estimate MU numbers the values probably only reflect numbers in an ill-defined volume of muscle and are not representative of the whole muscle. A method based on needle electrode recordings is described which provides an estimate that is more representative of the whole muscle and is suitable for use with large proximal muscles that are important for mobility but may deteriorate with ageing.

## Abbreviations

BB: Biceps brachii
CMAP: Compound muscle action potential
EMG: Electromyography
iEMG: Intramuscular electromyography
MU: Motor unit
MUP: Motor unit potential
MUNE: Motor unit number estimation
MRI: Magnetic resonance imaging
MVC: Maximal voluntary contraction
sEMG: Surface electromyography
sMUP: Surface motor unit potential
STA: Spike triggered averaging
TA: Tibialis anterior
VL: Vastus lateralis
